# Supplementary material for: Effect of Graphene Oxide on the Mechanical Properties and Durability of High-Strength Lightweight Concrete Containing Shale Ceramsite
Source: Materials (Basel). 2023 Mar 30;16(7):2756. doi: 10.3390/ma16072756 (PMC10095860; doi:10.3390/ma16072756)
Supplement: Supplementary file 1 [file materials-16-02756-s001.zip › materials-2267814-supplementary.pdf]

Effect of Graphene Oxide on the Mechanical Properties and Durability of High-Strength Lightweight Concrete Containing Shale Ceramsite

Xiaojiang Hong [1,2], Jin Chai Lee [1,*], Jing Lin Ng [1,2], Zeety Md Yusof [3], Qian He [1,2] and Qiansha Li [1,2]

[1] Department of Civil Engineering, Faculty of Engineering, Technology and Built Environment, UCSI University, Kuala Lumpur 56000, Malaysia
[2] Department of Civil Engineering, Faculty of Civil and Hydraulic Engineering, Xichang University, Xichang 615013, China
[3] Department of Civil Engineering, Faculty of Civil Engineering and Built Environment, Universiti Tun Hussein Onn Malaysia, Parit Raja 86400, Malaysia
* Correspondence: leejc@ucsiuniversity.edu.my or jinlee861@gmail.com

Formula (S1)	Formula of the chloride ion migration coefficient (D_{RCM})
Formula (S2)	Formula of mass loss rate of freezing–thawing resistance
Formula (S3)	Formula of the relative elastic modulus
Formula (S4)	Formula of mass loss rate of sulfate attack resistance
Formula (S5)	Formula of corrosion resistance coefficient

Supplementary Materials

1 Formula of chloride-ion migration coefficient (D_{RCM}):

$$D_{RCM} = \frac{0.0239\times(273+T)L}{(\mathrm{U}-2)\mathrm{t}}\left(X_d - 0.0238\sqrt{\frac{(273+T)LX_d}{U-2}}\right) \quad \text{(S1)}$$

where D_{RCM} is the unsteady transfer coefficient of concrete, accurate to 0.01×10^{-12} m^2/s; T is the average of the initial and final temperatures of the anode solution, °C; U is the test voltage, V; L is the thickness of the specimen, mm; X_d is the average chloride-ion penetration depth, mm; t is the test duration, h.

2 Formula of mass loss rate of freezing–thawing resistance:

$$\Delta W_{ni} = \frac{W_{0i}-W_{ni}}{W_{0i}} \times 100 \quad \text{(S2)}$$

where ΔW_{ni} is the mass loss rate of the ith specimen after n freeze–thaw cycles, %, accurate to 0.01; $W_{0i}$is the mass of the ith specimen before the freeze–thaw cycle, g; W_{ni} is hte mass of the ith specimen after n freeze–thaw cycles, g.

3 Formula of relative dynamic elastic modulus:

$$P_i = \frac{f_{ni}^2}{f_{0i}^2} \times 100 \quad \text{(S3)}$$

where P_i is the relative dynamic elastic modulus of the ith specimen after n freeze–thaw cycles, %, accurate to 0.1; f_{0i} is the initial value of transverse fundamental frequency of the ith specimen before the freeze–thaw cycle, Hz; f_{ni} is the test value of the transverse fundamental frequency of the ith specimen after n freeze–thaw cycles, Hz.

4 Formula of mass loss rate of sulfate attack resistance:

$$\Delta W'_{ni} = \frac{W'_{0i} - W'_{ni}}{W'_{0i}} \times 100 \quad \text{(S4)}$$

where $\Delta W'_{ni}$ is the mass loss rate of the ith specimen after n wet–dry cycles, %, accurate to 0.01; W'_{0i} is hte mass of the ith specimen before the wet–dry cycle, g; W'_{ni} is the mass of the ith specimen after n wet–dry cycles, g.

5 Formula of corrosion resistance coefficient:

$$K_f = \frac{f_{cn}}{f_{c0}} \times 100 \quad \text{(S5)}$$

where K_f is the corrosion resistance coefficient, %; f_{c0} is the compressive strength value of the specimen under standard curing at the same age as sulfate corrosion., accurate to 0.1 MPa; f_{cn} is the compressive strength value of the specimen corroded by sulfate after n times of dry and wet cycles, accurate to 0.1 MPa.
